# Encoding of antennal position and velocity by the Johnston's organ in hawkmoths

**DOI:** 10.1242/jeb.249342

**Published:** 2025-05-02

**Authors:** Chinmayee L. Mukunda, Sanjay P. Sane

**Affiliations:** National Centre for Biological Sciences, Tata Institute of Fundamental Research, GKVK Campus, Bellary Road, Bangalore 560065, India

**Keywords:** Mechanosensors, Chordotonal, Sensory encoding, Neurophysiology, Adaptation, Hysteresis, Intracellular, *Daphnis nerii*

## Abstract

Insect antennae function as versatile, multimodal sensory probes in diverse behavioural contexts. In addition to their primary role as olfactory organs, they serve essential mechanosensory functions across insects, including auditory perception, vestibular feedback, airflow detection, gravity sensing and tactile sensation. These diverse functions are facilitated by the mechanosensory Johnston's organ (JO), located at the joint between the flagellum and the pedicel (second antennal segment). This joint lacks muscles, which means that JOs can perceive only passive deflections of the flagellum. Earlier work that characterized the sensitivity and short response time of the JO sensory units in hawkmoths showed that their sensitivity to a broad frequency range is range-fractionated. This vastly expands the functional repertoire of the JO. However, it is not clear what components of antennal kinematics are encoded by the JO. Here, we conducted experiments to test the hypothesis that JO neurons encode the position and velocity of angular movements of the flagellum. We recorded intracellularly from the axons of primary sensory neurons of the JO while stimulating it with ramp-and-hold stimuli in which either the antennal position or antennal angular velocity was maintained at various constant values. Our study shows that JO neurons encode angular velocity and position of the antenna in their response. We also characterized the neural adaptation of the responses to angular velocities and positions. The majority of neurons were sensitive to a movement in the ventrad direction, in the direction of gravity. The adaptation and directional response properties give rise to a nonlinear hysteresis-like response. Together, these findings highlight the neurophysiological basis underlying the functional versatility of the JO.

## Teaser

**Summary:** The mechanosensory Johnston's organ (JO) in hawkmoth antennae encodes the position, velocity and direction of antennal movements in response to external stimuli, revealing the neurophysiological basis of JO's functional versatility.

## INTRODUCTION

Despite the staggering diversity in forms and structures, the antenna of ectognathan insects is a highly conserved multisensory organ, facilitating olfaction, thermoception, hygroception and mechanosensation across various species. In particular, the mechanosensors on the insect antennae mediate various important functions including tactile sensing (cockroaches; Camhi and Johnson, 1999; Okada and Toh, 2000; Comer et al., 2003; Comer and Baba, 2011), audition (mosquitoes, honeybees and flies; Johnston, 1855; Dreller and Kirchner, 1993a,b; Eberl, 1999) and vestibular feedback for balance during flight (Lepidoptera; Niehaus, 1981; Sane et al., 2007, 2010, 2023). Thus, the mechanosensory properties of the insect antenna provide a compelling model for investigating sensory encoding and its functional implications.

The antennae of all neopteran insects consist of three segments: a basal scape, a middle pedicel and a distal flagellum (Schneider, 1964; Krishnan and Sane, 2015). Whereas the head–scape and scape–pedicel movements are actuated by the extrinsic and intrinsic muscles, respectively, movements of the flagellum relative to the pedicel are passive and moved only by external perturbations or indirectly owing to body movements that cause inertial forces on the pedicel–flagellum joint (Kloppenburg et al., 1997; Sant and Sane, 2018). These passive flagellar deflections are detected by an extremely sensitive mechanosensory chordotonal organ, the Johnston's organ (JO), which spans across the pedicel–flagellum joint (Johnston, 1855; Gewecke, 1974). In many insects including cockroaches, various mechanosensors distributed along the flagellum are responsible for detecting its bending (Camhi and Johnson, 1999; Staudacher et al., 2005; Dürr et al., 2022). Additionally, diverse insects including moths, locusts and bugs possess other chordotonal organs and campaniform sensilla, in conjunction with the JO, which can also sense distortions at the pedicel–flagellum joint (Schneider and Kaissling, 1957; Heinzel and Gewecke, 1979; Jeram and Pabst, 1996; Staudacher et al., 2005).

JOs serve diverse functions across insects. For instance, honeybee JOs are thought to perceive the auditory cues generated by a forager bee during waggle dances (Dreller and Kirchner, 1993a,b), as well as airflow during flight (Heran, 1957; Roy Khurana and Sane, 2016). In mosquitoes, JO neurons encode the auditory stimuli generated during flight and courtship (Gopfert and Robert, 2001; Lapshin and Vorontsov, 2017). These mechanosensors are also involved in antennal positioning and providing flight-related feedback in blowflies, locusts, hawkmoths, bees and vinegar flies (Burkhardt and Gewecke, 1965; Gewecke, 1967; Sane et al., 2007; Mamiya and Dickinson, 2015; Roy Khurana and Sane, 2016; Suver et al., 2019), and tactile substrate vibration sensing in stink bugs (Jeram and Čokl, 1996). In hawkmoths, JOs provide vestibular feedback during flight (Sane et al., 2007, 2023; Dahake et al., 2018), airflow-dependent antennal positioning (Natesan et al., 2019), head stabilization (Chatterjee et al., 2022) and flower-tracking behaviours (Dahake et al., 2018). Together, this body of work demonstrates both the range and versatility of JO function. In all the above cases, JO is activated via the movement of the flagellum relative to the pedicel and hence its basic mechanism is conserved despite the diversity.

The JO is composed of several sensory units called scolopidia. Each scolopidial unit consists of dendrites of two to three sensory neurons that project to a scolopale cell that connects to the cuticular invaginations of the flagellum (Field and Matheson, 1998). Mechanoreceptors at the dendritic tip transduce the mechanical vibrations of the flagellum into receptor potentials (Zanini and Göpfert, 2014; Eberl et al., 2016). The cell bodies of the sensory neurons located in the pedicel integrate these receptor potentials and generate action potentials that encode the movements of the flagellum (Kamikouchi et al., 2009). This encoded information can be used by the nervous system as sensory feedback to modulate behaviours. To perceive subtle perturbations of the flagella during flight manoeuvres, the sensory neurons need to be sensitive to extremely small deflections and must respond very rapidly at high frequencies. Intracellular electrophysiological recordings in hawkmoth JO confirmed that sensory neurons respond to deflections of as little as ∼0.05 deg at latencies as low as 3 ms (Dieudonne et al., 2014). The JO is sensitive to a wide range of frequencies and range fractionated, which means that individual scolopidia do not trade off sensitivity and range, but are individually finely tuned to a narrow frequency range (hawkmoths, *Manduca sexta*; Sane et al., 2007; Dieudonne et al., 2014; mosquitoes, *Culex pipiens*; Lapshin and Vorontsov, 2017). Similar characteristics have also been observed through calcium imaging of JO neuron activity in *Drosophila* (Kamikouchi et al., 2009; Yorozu et al., 2009; Patella and Wilson, 2018).

Because mechanosensory neurons encode diverse mechanical stimuli across insects, the understanding of the fundamental features of the stimulus encoded by these neurons is crucial to determine the mechanisms that underlie the function. Specifically, what components of the ambient mechanical stimuli are encoded by JO neurons? Previous investigations of this question have used diverse stimuli including sinusoids, chirps and band-limited Gaussian noise stimuli to characterize the response to a wide range of stimulus frequencies (Sane et al., 2007; Dieudonne et al., 2014). These studies shed light on specific encoding properties of the JO neurons in relation to their function as antennal mechanosensors. However, an inherent limitation in the stimulus delivery system in these studies meant that the stimuli were mechanically filtered and hence, a readout of the delivered stimulus closest to the sensor of interest is essential for accurate interpretation of the results of these experiments. White noise stimuli that are commonly used in studying sensory encoding scrambles from the stimulus all of its natural context, which may render the resulting responses very difficult to interpret. Indeed, this lacuna is typical of any ‘systems level’ white noise characterization of sensory encoding properties. Moreover, a white noise analysis assumes the system to be stationary, whereas most mechanosensors exhibit adaptation. Besides such conceptual challenges, delivering truly white noise presents a methodological challenge.

An alternative approach involves examining the system's response to individual components of a complex stimulus. This method aims to gain insights from the system's reactions to simpler stimuli, which can then aid in understanding its responses to more complex stimuli. Using this latter approach, we presented stimuli designed to address the encoding properties of JO neurons. Specifically, we proposed that the neural activity of JO neurons encodes the angular position and velocity of flagellar movement, which represent the initial terms in the series expansion of any complex stimulus time series function. We presented the antenna of the oleander hawkmoth, *Daphnis nerii*, with either constant position or constant velocity stimuli, relevant in sensing static and low frequency stimuli such as gravity and airflow, respectively. Simultaneously, we performed intracellular recordings from axons of JO neurons in the antennal nerve. Here, we describe the encoding properties of JO neurons as they relate to components of a movement stimulus. We also discuss the similarities observed in the physiology of the JOs with femoral chordotonal organs and campaniform sensilla in the legs of insects, suggesting common underlying encoding principles across these mechanosensors.

## MATERIALS AND METHODS

### Moth culture

Wild oleander hawkmoth [*Daphnis nerii* (Linnaeus 1758)] larvae or pupae initially collected from the surrounding area were bred in a large cage housing host plants and artificial nectaries. The female moths lay eggs on the leaves of *Nerium oleander* or *Tabernaemontana divaricata* plants after mating. The eggs were collected in plastic containers. The hatched larvae were provided with tender *Nerium* leaves. The larvae were transferred into larger boxes with harder leaves as they grew old. *Daphnis nerii* undergoes five molts or ‘instars’ in the larval phase. Fifth instar larvae were kept in sawdust for pupation, which provided sufficient friction to remove their pupal case during emergence. After ∼15 days of pupal stage, the late-stage pupae were transferred into an emergence cage from which the moths were collected for experiments.

### Preparation

Adult *D. nerii* were used for all the experiments. Healthy male or female moths were collected from the moth culture facility 0–1 day post-eclosion. The moths were visually inspected for any deformities in the body and for normal activity levels to assess that they were healthy and active.

#### Immobilization

**Fig. 1.**
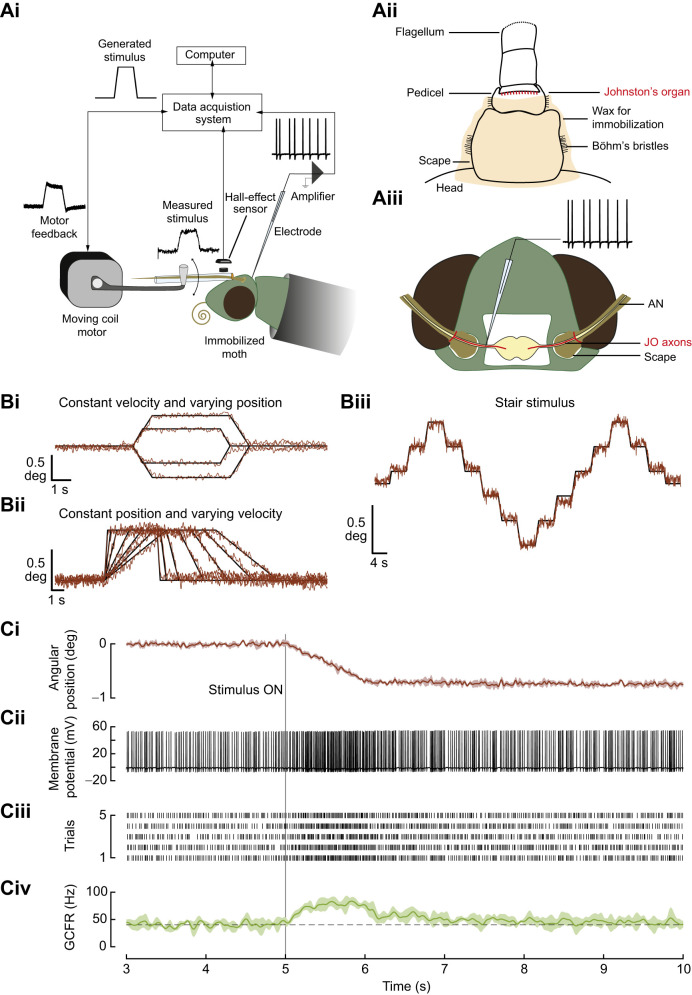
**Experimental setup and processing of raw data.** (Ai) Schematic of the experimental setup for intracellular recording from axons of single neuronal units of the antennal mechanosensory Johnston's organ (JO). A custom-made capillary attachment connects the flagellum to the motor. A Hall-effect sensor placed opposed to a neodymium magnet mounted on the capillary records the actual movement delivered. (Aii) The head–scape and scape–pedicel joints of the antenna are immobilized using a mixture of beeswax and rosin (3:2). This ensures that the stimulus acts only on the pedicel–flagellum joint where the JO is located. (Aiii) Schematic of the surgery performed to expose the moth brain and the antennal nerve. The axons of JO (in red) neurons project to the brain through the antennal nerve. The recording electrode is inserted into the antennal nerve (AN). (B) Comparison of computer-generated stimulus and the filtered output of the Hall sensor. Ramp-and-hold stimuli with (Bi) constant angular velocity and variable angular positions, (Bii) constant angular position and variable angular velocities and (Biii) constant velocity and constant relative change in position (stair protocol). (C) Analysis pipeline showing the processing of raw data. (Ci) Ventral ramp-and-hold stimulus delivered to the flagellum, as measured by the Hall-effect sensor. Solid line, mean of five trials; shaded region, 1 s.d. The grey line labeled ‘Stimulus ON’ marks the start of the stimulus following a rest period. (Cii) A representative trace showing a single trial of recorded membrane potential in response to the above stimulus. Action potentials (or spikes) with hyperpolarization are indicative of intracellular recording. (Ciii) Raster plot showing spike timing in five trials of the same stimulus. (Civ) Gaussian convolved firing rate (GCFR) is a smoothed histogram of firing rate (mean±s.d., solid line and shaded region, respectively); the dashed line marks the average baseline firing rate.

The head, neck, thorax and wing bases were descaled using a toothbrush for proper immobilization. The legs were detached at the coxa–trochanter joint to minimize haemolymph loss. The wings, thoracic sclerites, thorax–abdomen joint and head–neck joint were immobilized using hot dental wax. The moth was placed dorsal side up in a customized plastic syringe tube and attached to the tube using hot wax; the dorsal side of the head was kept accessible for antennal stimulation and surgery (Fig. 1Ai).

#### Positioning antenna for stimulus

The tube holding the moth was mounted on a stand in an electrophysiology rig at an angle of ∼40 deg. The flagellum of the left antenna was passed through a custom-made capillary, which was attached to the motor shaft. The antenna was positioned at ∼45 deg to the longitudinal axis of the moth. The head–scape and scape–pedicel joints of the left antenna were immobilized (Fig. 1Aii) by depositing a mixture of beeswax and rosin (3:2), using a custom-made cauterizer with fine filament (∼0.5 mm tip). Rosin (also known as colophony) is a resin added to beeswax to make the mixture hard so that the deposited mixture can maintain structural integrity. The two joints were immobilized to specifically stimulate JO and restrict the activity of the antennal musculature. The flagellum of the left antenna was passed through a custom-made capillary and attached to it at the third annulus using a beeswax mixture. This ensured that when the capillary moved, the flagellum did not slide within the capillary. The glass capillary, in turn was attached to the motor shaft. The immobilization of the head–scape and scape–pedicel joints and, affixing the flagellum to the capillary attachment in this manner, ensured that only the JO mechanosensors at the pedicel–flagellum joint were stimulated. All the joints of the right antenna were immobilized with wax to minimize movements in the preparation.

#### Surgery

The cuticle of the head and the tissue underneath were cut out to expose the brain and the antennal nerve (Fig. 1Aiii). The trachea on the nerve were removed using fine forceps for easy penetration of the nerve by the electrode. The antennal nerve was supported from underneath by a bent syringe needle (PrecisionGlide^®^ needle, 305128). The tip of the needle was coated with a resin-based adhesive (Araldite^®^) and dried, thus eliminating slippage of the nerve and providing stability for recordings. This needle also delivered insect saline (recipe below) to the preparation.

### Electrophysiology

Intracellular recordings were performed on individual axons of the JO neurons passing through the antennal nerve. The exposed region was constantly perfused with physiological insect saline. The saline consisted of 150 mmol l^−1^ NaCl, 3 mmol l^−1^ CaCl2, 3 mmol l^−1^ KCl, 10 mmol l^−1^ N-tris (hydroxymethyl) methyl-2-aminoethane sulfonic acid buffer and 25 mmol l^−1^ sucrose (Lei et al., 2004; Dieudonne et al., 2014); pH was adjusted to 6.9 using NaOH. Chloridized silver wires were used as recording and reference electrodes. The reference electrode was placed in the vicinity of the antennal nerve and dipped in the saline perfusion. A quartz capillary (outer diameter: 1 mm, inner diameter: 0.5 mm, with filament, Sutter Instruments, Novato, CA, USA) was pulled to standardized dimensions using a customized program in the pipette puller (P-2000, Sutter Instruments). The recording electrode was placed in the pulled pipette filled with 1 mol l^−1^ KCl. The tip of the electrode broke after touching the nerve sheath, following which bridge balancing was performed. The resistance of the electrodes before recording were ∼100 MΩ.

The recording electrode was connected to the amplifier head stage through a 3D-printed custom electrode holder. The head stage was mounted on a micromanipulator (EMM2, Narishige, Japan) that controlled the precise position of the electrode. The electrode was gradually lowered into the antennal nerve to record activity of individual JO neurons. As described above, care was taken to stimulate only the pedicel–flagellum joint. However, we cannot rule out the possibility of non-specific effects of the stimulus such as strain or torsion on the JO, or that other mechanosensors along the length of the antennae may be occasionally stimulated. The recorded activity was amplified with a gain of 10 (IX2 pre-amplifier, Dagan Corporation, Minneapolis, MN, USA). All the signals were registered in the computer through the data acquisition system (USB-6363, National Instruments, Austin, TX, USA) (Fig. 1Ai).

### Stimulus delivery

The capillary enclosing the flagellum was connected to a motor (300C-I, Aurora Scientific, Canada) through a linkage system (Fig. 1A). This system was driven by computer commands delivered through the output channel of the data acquisition system. The stimulus protocols were generated by a custom-written MATLAB program (MathWorks, Natick, MA, USA), available at https://github.com/chinmayeelm/Intracellular-recording-Matlab-app.git.

The actual stimulus experienced by the antenna is considerably different from the intended stimulus, as it is altered by the mechanical apparatus linking the motor to the antenna. It is thus necessary to measure the actual stimulus, especially when working with highly sensitive mechanosensors. The stimuli delivered in all our experiments were along a single axis (dorsoventral). To obtain a readout of the actual stimulus delivered to the JO, a neodymium magnet (diameter=3 mm, thickness=2 mm) was mounted close to the tip of the capillary holding the flagellum. A Hall-effect sensor aligned with the magnet was used to measure the change in the magnetic field as the flagellum moved upon stimulus delivery. The Hall-effect sensor thus provided a more faithful readout of the actual stimulus delivered, closer to the base of the antenna. Although the values mentioned below indicate the intended stimuli (angular displacements and angular velocities), all the analyses and related plots were obtained using the actual readouts from the Hall-effect sensor as stimuli. A voltage versus distance calibration curve obtained for the sensor-magnet pair provided the actual distances by which the capillary moved. The distance between the third annulus and the pedicel–flagellum joint was measured in every moth at the end of the experiment session under a microscope (SMZ25, Tokyo, Japan) with calibrated scale in the associated software (NIS-Elements). The actual angular movements delivered to the JO were calculated using the formula:
(1)

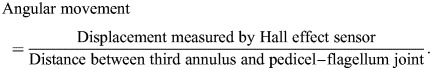


### Stimulus protocols

The MATLAB-generated stimulus protocols were designed to obtain both the static and dynamic responses of the neurons (Fig. 1Bi–iii). A step stimulus has been typically used to estimate the response latencies, adaptation dynamics and encoding properties of mechanosensors (Chapman and Smith, 1963; Dieudonne et al., 2014). An ideal step stimulus involves an instantaneous change in position, which is not possible for a mechanical system to achieve. The neuron may respond to the sudden deflection in position, velocity or acceleration of the movement, making it difficult to identify and delineate which components of the delivered stimulus elicited the response. To overcome this issue, we used ramp-and-hold stimuli in which angular velocities and angular positions could be independently varied. This allowed us to decouple the response of neurons to angular velocities and positions. Similar stimulus protocols have been used in the physiological characterization of femoral chordotonal organs (Hofmann et al., 1985), campaniform sensilla (Ridgel et al., 2000) and mechanosensory descending neurons (Ache and Dürr, 2013).

In the experiments described here, the range of hold positions tested was limited by how much the flagellum could be moved without affecting the stability of the recording. Besides, previous free-flight studies suggest that the flagellum undergoes small amplitude deflections (Sane et al., 2007). Owing to the limitations of the motor, there was also a trade-off between the amplitude and velocity of movement. Hence, it was not possible to attain the same hold positions exceeding two orders of magnitude change in constant angular velocity.

The protocols were as follows: ramp-and-hold stimuli with variable hold positions; ramp-and-hold stimuli with variable ramp velocities; and stair.

#### Ramp-and-hold stimuli with variable hold positions

The flagellum was moved to different positions (1 or 0.5 deg above and below baseline position) at a fixed angular velocity of 1 deg s^−1^ (Fig. 1Bi).

#### Ramp-and-hold stimuli with variable ramp velocities

The flagellum was moved by 1 deg at different velocities (0.1 to 4 deg s^−1^) and held at constant position for 4 s (Fig. 1Bii).

#### Stair

A stair protocol spanned 2 deg in steps of 0.4 deg. The transitions between the positions were made at a constant velocity of 0.04 deg s^−1^ in both dorsal and ventral directions. Each step lasted for 4 s (Fig. 1Biii).

**Fig. 2.**
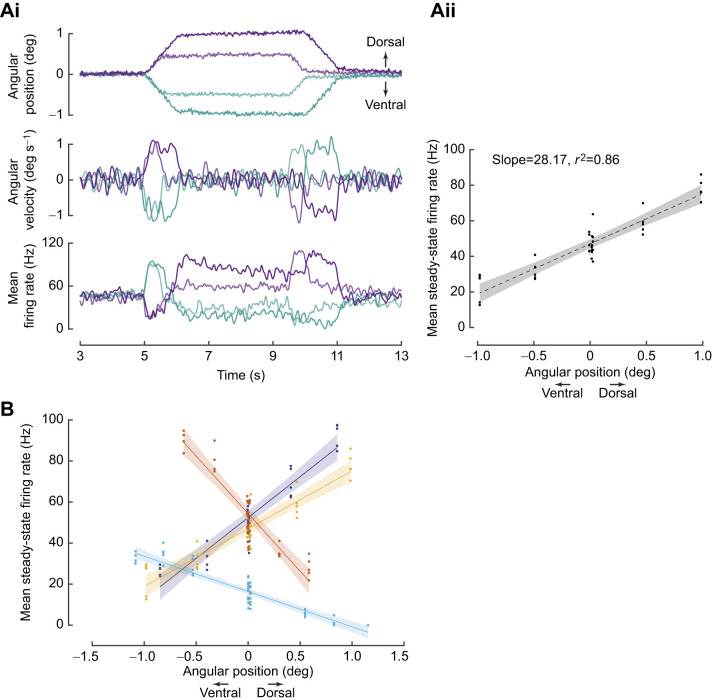
**Position-encoding neurons.** (A) A neuron sensitive to both angular position and velocity. (Ai) Top: mean angular position stimulus delivered to the flagellum (five trials per protocol). The flagellum was deflected by ca. 0.5 and 1 deg above and below baseline. Middle: angular velocity profile of the stimulus shows the same magnitude of velocity during the ramp phases to both positions in the same direction. Bottom: mean firing rate obtained from five trials for each protocol. (Aii) Mean steady-state firing rate is measured as the mean firing rate over 2.5 to 3.5 s of the constant position stimulus. The steady-state firing rate linearly increased with angular position. Dashed line, linear fit; shaded region, 95% confidence interval. (B) Steady-state firing rate response to constant angular positions in four out of 20 neurons that showed a linear relationship with position with *r*^2^≥0.8.

**Fig. 3.**
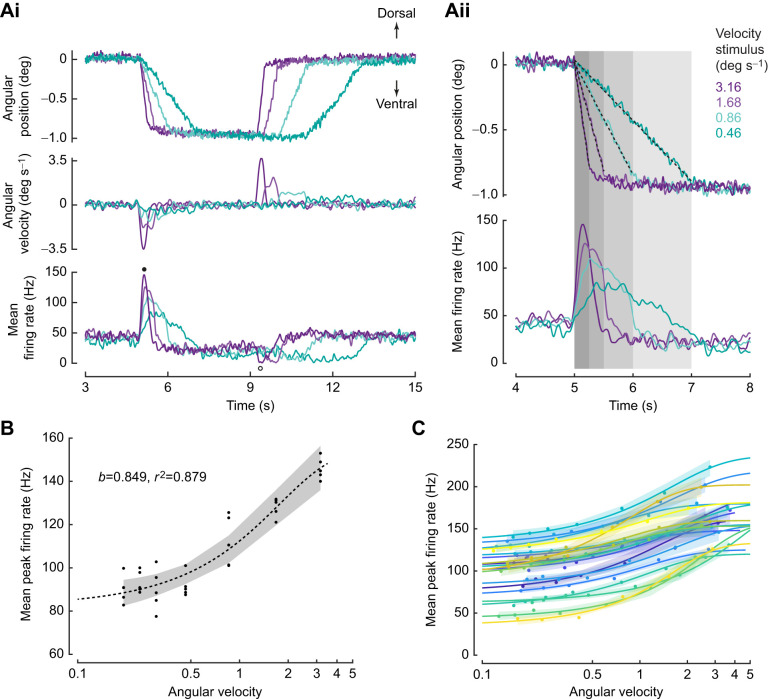
**Velocity-encoding neurons.** (Ai) Top: mean angular position stimulus delivered to the flagellum (five trials per protocol). The velocity stimulus varied from 0.2 to 3.1 deg s^−1^ (four out of seven protocols shown for clarity; blue–green to purple traces with velocities: 0.46, 0.86, 1.68, 3.16 deg s^−1^). Middle: angular velocity profile. Bottom: average firing rate obtained from five trials for each velocity stimulus. Increase and decrease in firing rate corresponding to ventral and dorsal ramp movement are marked by closed and open circles, respectively. (Aii) A detailed view of the peak firing rates during the constant angular velocity stimuli. The grey boxes mark the duration of the ramp stimulus. The dashed black lines are the linear fit performed on the ramp to estimate angular velocity. (B) Mean peak firing rate response to angular velocity (deg s^−1^) is modelled by a logistic function. Dashed line, logistic fit; shaded region, 95% confidence interval. The *x*-axis is on a log scale. (C) In 19 out of 27 neurons, *r*^2^≥0.8. The solid lines are the logistic fits for individual neurons and shaded regions show the 95% confidence interval of the fit. The *x*-axis is on a log scale.

A resting period of 6–10 s (corresponding to approximately 180–300 wing beats) was provided between consecutive trials, allowing the neural activity to return to the baseline level. Positive and negative values of angular position or velocity correspond to a dorsal or a ventral movement of the flagellum, respectively. The effective values of angular positions and velocities depended on the point of attachment of the flagellum to the capillary, which varied across preparations. Nevertheless, the stimulus presentations were reliably repeatable within recordings from individual moths. For example, if the actual deflection was 0.8 deg when the target deflection was 1 deg, this value was consistently achieved across all trials and protocols tested on that moth. The angular velocity profile of the stimulus signal is displayed in the middle panels of Figs 2A and 3A (also Fig. S2A,C). The velocity signal was obtained by taking the derivative of the position signal from the Hall-effect sensor and filtering with a lowpass Butterworth filter (order=3, cutoff=4 Hz). This filtering causes the peaks to be attenuated. Thus, the reported values of velocities do not match the peak of the velocity signals in these plots. To obtain a reliable estimate of the velocity of the ramp, we fit a straight line to the ramp. The *r*^2^ of these fits was always greater than 0.9. The slope of the fit is the reported constant angular velocity value during the ramp stimulus.

### Data analysis

For all the analyses presented here, we have used only recordings with at least three trials per protocol. All the data analyses were performed in MATLAB.

#### Spike detection

As described above, the prescribed stimulus was transformed by the mechanical system, and hence we used a Hall-effect sensor to record the actual stimulus close to the antennal base (Fig. 1Ci). The recorded intracellular response was bandpass-filtered with a Butterworth filter (order=8, cutoff=10–2000 Hz) to eliminate drifts, baseline offsets and high-frequency noise. Thirty percent of the maximum amplitude of membrane voltage within a trial was set as the threshold for spike detection (Fig. 1Cii). The spike timings across the trials were collated to generate a raster (Fig. 1Ciii). Trials with membrane voltage lower than 5 mV were discarded, as the small amplitude spikes may be indistinguishable from extracellular spikes from adjacent neurons.

#### Gaussian convolved firing rate (GCFR)

A smoothed firing rate was obtained by convolving the raster data with a Gaussian kernel of 200 ms width and 30 ms standard deviation (Fig. 1Civ).

#### Baseline firing rate

The baseline firing rate is the firing rate before the start and end of a stimulus protocol. Most neurons showed some baseline activity during the resting periods while the flagellum is held at a constant angle. For instance, the neuron shown in Fig. 1C had a mean baseline firing rate of 40 Hz which increased to a maximum of 83 Hz, when the antenna was moved ventrally and then fell to 46 Hz when the antenna was again at a new steady position of 0.7 deg (Fig. 1Civ).

#### Definitions

The following definitions apply to the recordings and analysis in this paper. Mean baseline firing rate is the mean firing rate calculated over 1 s duration of the trial-averaged firing rate, preceding the onset of the ramp stimulus by 0.5 s. Peak firing rate is the maximum firing rate attained within the duration of the ramp stimulus. Steady-state firing rate is the firing rate attained after the adaptation to constant position stimulus. Mean steady-state firing rate is the mean firing rate calculated over 1 s duration of the trial-averaged firing rate, preceding the end of hold stimulus by 0.5 s.

#### Statistical tests

Spearman's correlation was used to test the correlation between angular position and steady-state firing rate, and a linear fit was used to further describe the relationship. A sigmoidal fit was used to describe the relationship between peak firing rate and angular velocity.

##### Quantifying the relationship between the constant angular velocity stimulus (ramp) and corresponding peak firing rate

To understand how peak firing rate relates to angular velocity, we tested four models from the literature and compared how well each one explained the data. Details of these four models are given in the Supplementary Materials and Methods, with plots in Fig. S1 along with a table of fit parameters (Table S1). In three out of 30 neurons, the response to ramp stimulus was not distinguishable from baseline. These were excluded from this analysis.

Various studies in the past have used power law and semilog fits to model the relationship between firing rate and the first-order derivative of a stimulus (Matheson, 1992; Ridgel et al., 2000; Zill et al., 2011). Technically, two orders of magnitude change in stimulus parameter and firing rate are required to establish a power law relationship. Although these models fit our data well, because the sampled velocity range was less than two decades, we could not justify using either power law or semilog fits. These models were nevertheless applied for comparison with previous literature, and are included in the Supplementary Materials and Methods. Of the four models, the logistical model provided the best fit (*r*^2^≥0.9) for most neurons, and hence this was adopted for all analysis.

##### Logistic fit

Some neurons showed a saturating nonlinear response to angular velocity. We modelled the nonlinear relationship as a logistic (sigmoidal) equation (Landolfa and Miller, 1995):
(2)

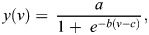
where *y*(*v*) is the peak firing rate, *v* is the angular velocity of flagellar movement, and *a*, *b* and *c* are coefficients of the equation.

##### Quantifying adaptation

The ramp-and-hold stimulus is composed of a constant velocity and a constant position segment. To study the adaptation to each of these stimuli, we separately extracted the responses to the two stimuli. We quantified the adaptation to constant angular velocity (or ramp) stimulus by extracting the response from 90% of the maximum firing rate to the end of the ramp stimulus time point. To quantify adaptation to the hold stimulus, the response 100 ms after the end of the ramp stimulus to 500 ms before the end of the hold stimulus was considered. Single decaying exponential fits have been commonly used to model adaptation in neurons (Clemens et al., 2018; Hildebrandt et al., 2009). Here, we modelled the adaptation of the firing rate with two separate single exponential fits:
(3)

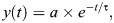
where *y*(*t*) is the firing rate, *t* is time, τ is the adaptation time constant and *a* is the scaling coefficient. τ*>*0 implies adaptation, whereas τ<0 implies an increasing response.

In many cases, the *r*^2^ values were low owing to fluctuations in the firing rate. Only fits with *r*^2^≥0.75 were considered for further analysis.

## RESULTS

The data from all the recorded neurons (60 neurons, from 32 moths) and their characteristics are summarized in Table 1. Neuronal recordings used as specific case examples are highlighted wherever relevant (Table S2). Most neurons showed some baseline activity during the resting period when the flagellum was held in a constant position, indicating the presence of residual deformation in the joint. In 56 out of 60 neurons, the firing rate increased relative to the baseline when the flagellum was moved in the ventral direction. In the remaining four neurons, the response was ambiguous and therefore excluded from the analysis. To understand how the neuronal response encodes angular positions and velocities, we used ramp-and-hold stimuli in which either the hold position (i.e. angular position) was varied at constant ramp slope, or the ramp slope (i.e. angular velocity) was varied at constant hold positions. Below, we describe activity of neurons encoding angular position and angular velocity, as well as exhibiting adaptation, history-dependence and directional sensitivity.

**Table 1.** Summary of responses of all position-, velocity- and direction-sensitive neurons

Response type	No. of neurons (moths)
Ventral velocity sensitive	19/27 (26)
Ventral hold position and ventral velocity sensitive	2/20 (18)
Dorsal hold positions sensitive and ventral velocity	1/20 (18)
Sensitivity to dorsal hold positions and the start of ventral ramp movement	1/30 (26)
Sensitivity to onset and offset of movement in either direction	1/30 (26)
Ventral ramp movement	56/60 (32)
Dorsal ramp movement	1/60 (32)
Dorsal hold positions	3/20 (18)
Ventral hold positions	2/20 (18)

Responses of nine neurons were excluded as they did not significantly change their activity in response to velocities, positions or ramp movements in either direction.

### JO neurons encode angular position of the flagellum

We moved the flagellum along a ramp-and-hold pattern to different positions at the same velocity (Fig. 2Ai, top and middle panels). This stimulus was presented to 20 neurons, of which 18 exhibited an initial rise or fall in firing rate during the ramp phase, depending on the direction of the ramp. This was followed by an easing (or adaptation) in firing rate during the hold phase, or sometimes even prior to onset of the hold phase. In the remaining two neurons, the response to the ramp stimulus was ambiguous; in one case, the GCFR was noisy with no apparent trend, and in another, the firing rate decreased and increased within the duration of ventral ramp movement.

For instance, in the neuron shown in Fig. 2A**,** the firing rate increased during ventral ramp movement (blue–green trace) and decreased during dorsal ramp movement (purple trace) (Fig. 2Ai, bottom panel). Thus, this neuron showed directional sensitivity. Moreover, the neuron responded with higher firing rates for dorsal hold position with slight adaptation, and lower firing rates for ventral hold positions (also see Fig. S2A). During the hold phase, the mean steady-state firing rate varied linearly as the angular position at which the antennae were held (Spearman's rho=0.86, linear fit *r*^2^≥0.8; Fig. 2Aii), demonstrating their sensitivity to angular position (also Fig. S2B).

In six out of 20 recordings (*N*=18 moths), the steady-state firing rate showed a strong correlation with angular position of the flagellum (Spearman's correlation, ρ>±0.8). In four out of these six neurons, the firing rate of JO neurons varied linearly with angular position of the flagellum (Fig. 2B). Of these, two neurons increased their firing rate, and two decreased their firing rate in response to varying dorsal hold positions of the flagellum. In our experiments, neurons were not labelled, and hence we could not determine their position within the JO relative to the stimulus, or whether dorsal or ventral ramp movements corresponded to stretch or compression of the JO mechanoreceptors. In the remaining 16 neurons, the firing rates during the hold phase did not show a linear relationship with position.

### JO neurons encode angular velocity of the flagellum

We next examined whether JO neurons were sensitive to angular velocity of the flagellum by subjecting them to a protocol in which the flagellum was moved to the same angular position, albeit at variable angular velocities broadly ranging from as low as ∼0.1 deg s^−1^ to as high as ∼4 deg s^−1^.

Fig. 3A shows recordings in response to the above protocol for the same neuron as in Fig. 2. Here, the antenna was subjected to seven different angular velocities, of which four representative stimuli and their responses are shown (Fig. 3A). In these four stimuli, the angular displacement was ∼0.7 deg but the angular velocities were 0.46, 0.86, 1.68, 3.16 deg s^−1^, respectively (Fig. 3A, top and middle panels). We estimated their angular velocity by fitting a line through the ramps at the beginning of the stimulus (Fig. 3Aii, top panel; see Materials and Methods for the angular velocity estimation). This neuron fired at a maximum rate (∼145 Hz) for the maximum angular velocity presented (∼3.16 deg s^−1^, purple trace), and at proportionally lower rates for the lower angular velocities (Fig. 3Ai, bottom panel; magnified in Fig. 3Aii, bottom panel). Thus, these neurons were sensitive to the angular velocity of the flagellum. We employed four different models to ascertain the relationship between the peak firing rate and angular velocity (details in the Supplementary Materials and Methods). Based on this comparison, we chose a logistic fit as the most appropriate model to explain the relationship for 19 out of 27 neurons (*r*^2^≥0.8; Fig. 3C), including the neuron shown in Fig. 3A (*r*^2^=0.88; Fig. 3B).

Ventral (downward) ramp movement of the flagellum increased the firing rate (closed circle), whereas dorsal (upward) ramp movement decreased the firing rate (open circle), thus demonstrating directional sensitivity of the JO (Fig. 3Ai, bottom panel).

### JO neurons exhibit adaptations to angular position and velocity

When the neuronal activity of a sensor decreases with time for a constant stimulus, the phenomenon is termed ‘adaptation’ (Adrian and Zotterman, 1926; Thorson and Biederman-Thorson, 1974; for a more recent review, see Benda, 2021). Typically, this decrease is exponential, and may be characterized using the time constants of these exponential decays (see Materials and Methods).

**Fig. 4.**
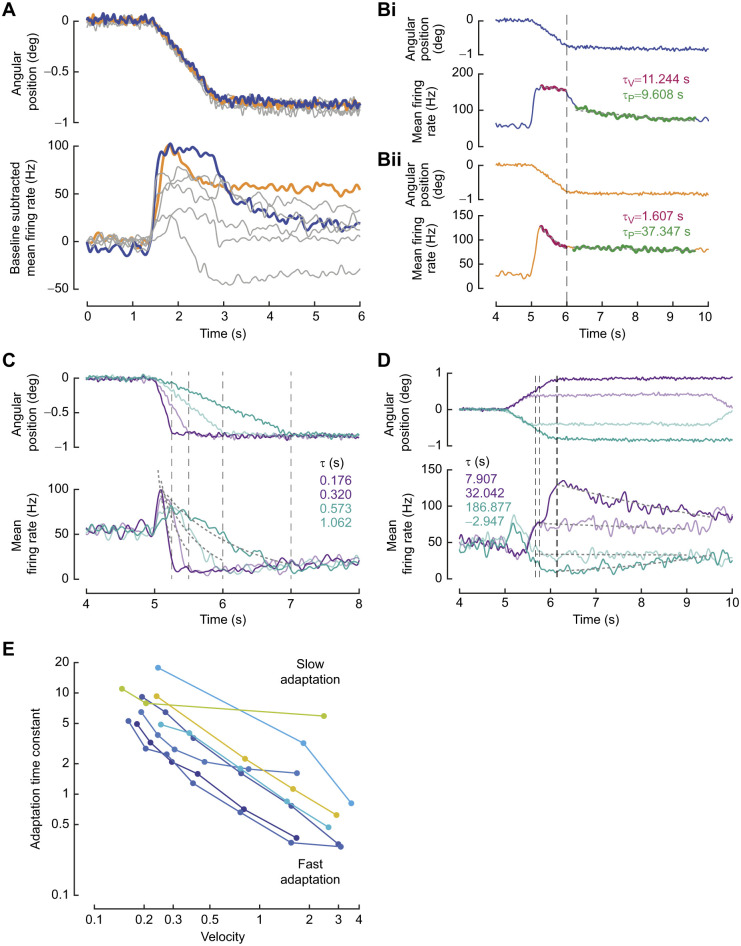
**Neurons exhibit adaptation to angular velocity and position.** (A) Responses of seven neurons to similar stimuli with steady-state positions of −0.84±0.03 deg (mean±s.d.), showing a wide range of adaptation dynamics exhibited by JO neurons. Responses of two neurons are highlighted in blue and orange for comparison. The baseline firing rate is subtracted for the ease of visual comparison of the nature of adaptation. Here, the ramp stimulus starts at 1.5 s. (B) Adaptation is quantified as the time constant of the exponential decay of the firing rate. The response of the two neurons highlighted in A, retaining the colour code. The dashed line marks the end of the ramp stimulus and the start of the hold stimulus. (Bi) Top: ramp-and-hold stimulus. Bottom: response of a neuron that shows slower adaptation to angular velocity but faster adaptation to constant position. (Bii) Top: ramp-and-hold stimulus. Bottom: response of a neuron that shows fast adaptation to velocity and slow/no adaptation to constant position stimulus. (C) Exponential fits and adaptation time constants to responses for four different angular velocities for the neuron in Fig. S2. Fits are shown with grey dashed lines (*r*^2^>0.75). (D) Exponential fits and adaptation time constants to responses for four different angular positions for the same neuron in C. Fits are shown with grey dashed lines (*r*^2^>0.5). (E) Responses of eight neurons (on a log–log scale) show a decrease in adaptation time constant (s) with an increase in angular velocity (deg s^−1^). Eight out of 26 neurons remained after eliminating the trials that had a fit with *r*^2^<0.75 and neurons that had less than three quantifications of time constant or positive time constants.

JO neurons exhibited diverse adaptation dynamics during the ramp-and-hold protocols. Fig. 4A shows the responses of seven different neurons to similar stimuli. The inherent adaptation of the responses to these stimuli varied widely. These responses are baseline subtracted for the ease of visual comparison. For instance, the two responses highlighted in Fig. 4A (bottom panel, blue and orange traces) show different adaptation dynamics to ramp movement and hold position. We quantified the adaptation in response to the two parts of the ramp-hold stimulus with two separate single exponential fits (see Materials and Methods) (Fig. 4B). τ denotes the adaptation time constant. Note that a lower value of τ indicates faster adaptation, and a larger τ indicates slower adaptation. The subpanels Fig. 4Bi,ii present data from two different neurons that adapted to angular velocities (ramp phase) and positions (hold phase) to different extents. The blue trace shows a slow adaptation to constant angular velocity compared with the orange trace, captured by an adaptation time constant of 11.2 versus 1.6 s (Fig. 4Bi,ii, highlighted in pink). During the hold position stimulus, the blue trace adapts faster than the orange trace, captured by an adaptation time constant of 9.6 versus 37.3 s (Fig. 4Bi,ii, highlighted in green). Thus, individual JO neurons had different adaptation properties that determined their response dynamics.

Fig. 4C shows the adaptation of firing rate for the neuron that linearly encodes angular positions (Fig. S2) during the ramp stimuli for constant hold position and varying angular velocities. Fig. 4D shows the adaptation during varying hold positions, while the ramp velocity was held constant. The values of adaptation constants in these protocols show that even within a single neuron, the adaptation dynamics varied with the value of angular velocity and angular position of the flagellum, i.e. adaptation was faster for larger values of angular velocity and position. As the angular velocity reduced, the firing rate took longer to adapt to the baseline firing rate, as reflected in greater time constants (Fig. 4C, bottom panel). For larger angular deflections, the adaptation time constants were smaller, recapitulating faster adaptation. During the hold stimuli, the adaptation was slower for positions closer to the baseline. An adaptation of the firing rate towards the baseline firing was also observed for the most ventral hold position (Fig. 4D). Across eight neurons, the time constant consistently reduced with an increase in angular velocity (Fig. 4E).

### JO neurons are direction-sensitive and history-dependent

**Fig. 5.**
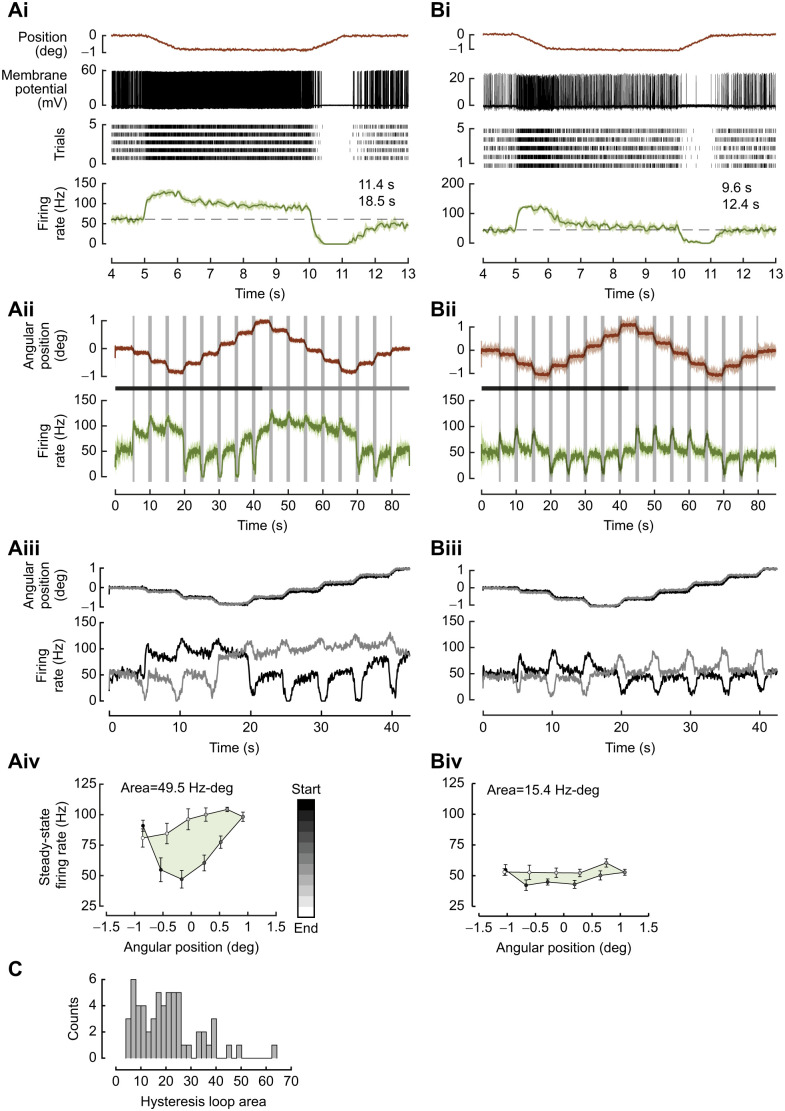
**Direction-sensitive responses of two neurons with low and high hysteresis.** (Ai,Bi) Top to bottom: ramp-and-hold stimulus; membrane potential of the first trial showing absence of spikes during dorsal ramp movement of the flagellum; raster plot showing response in five trials; mean±s.d. firing rate. Note the increased spike density in response to ventral ramp movement and the stark absence of spikes during dorsal ramp movement; the response shows a peak firing rate for ventral ramp movement, an adapting firing rate during the hold phase, and reaches a firing rate of 0 during dorsal ramp movement. Adaptation constants for adaptation of response to constant angular velocity are in black and constant angular position are in grey. (ii) Top: stair stimulus protocol (see Materials and Methods). Bottom: firing rate across trials (mean±s.d.). Grey bars mark the ramps between hold positions. (iii) Same as ii with the stimulus and response reversed and replotted such that the upstairs and downstairs stimuli overlap, to compare the responses to instances of the same positions within the protocol. In black, the stimulus and response from the beginning of the stimulus protocol to the midway point (shown with a black line below the stimulus trace in i). In grey, the stimulus and response from the midway point to the end of the stimulus protocol (shown with a grey line below the stimulus trace). (iv) Hysteresis in mean steady-state firing rate (mean±s.d.) response to similar angular positions. The colour of the circle marks the sequence of positions followed in the stimulus protocol. The initial and final three hold positions are not considered in the quantification of the hysteresis. The value in the top left is the area of the hysteresis loop. (C) Histogram of the area of the hysteresis loop from 59 neurons, showing that JO neurons exhibit a wide range of hystereses.

A prominent feature in most neurons was their sensitivity to the direction of ramp movement. Because the movement stimulus in the experiments reported here was uniaxial, the directional responses recorded here were restricted to the dorso-ventral movement of the flagellum. When stimulated with the stair protocol, 55 of 59 recorded neurons exhibited increased activity exclusively to either dorsal or ventral ramp flagellar movement, indicating a strong directional preference. Opposite to the preferred direction, the firing rate decreased. Such a response reversal was observed whenever stimulus changed direction in these 55 of 59 neurons (e.g. Figs 2A, 3A, 5Ai,Bi; Fig. S2A,C). In the remaining four neurons, the response was ambiguous owing to variability in the response. Moreover, in some neurons, this directionality was exhibited for both ramp movement and hold position in opposite directions. For example, in Fig. 2 and Fig. S2A, neurons showed a preference towards ventral ramp movement, but dorsal hold positions. Because these recordings were from primary mechanosensory neurons with low latency responses, the reduced response likely reflects a reduction in spiking activity (membrane potential and raster plots in Fig. 5Ai,Bi) rather than active inhibition.

Studies on the femoral chordotonal organ have shown inhibitory inputs from campaniform sensilla and the coxal hair plate (e.g. Stein and Schmitz, 1999). Efferent neuromodulatory receptors have been found in mosquito JOs (Andrés et al., 2016), suggesting a possibility that inhibitory inputs may arrive from the brain on to JO afferents. However, antennal nerve fills or JO fills in *D. nerii* have not revealed any projections on to the JO neurons (Sant and Sane, 2018; M. Srinivasan, G. Kondakath and S.P.S., unpublished data).

To observe how direction sensitivity influenced the response to constant position stimuli, we designed a stair protocol in which the antenna was held at various positions while keeping constant the step size and the angular velocity during the transition. Here, the same hold positions were attained during dorsal and ventral excursions. We recorded response of 59 neurons from 32 moths to the stair protocol. For instance, Fig. 5Aii and Bii show the response of two neurons to the stair protocol. In both neurons, there is a brief increase in activity during every ventral ramp movement, and a brief decrease during every dorsal ramp movement. The response adapted to the steady-state firing rate when hold position was constant (Fig. 5Ai,ii,Bi,ii).

Reversing and replotting the responses to upstairs and downstairs stimuli with overlapping hold positions (Fig. 5Aiii) shows that the steady-state firing rate encoding the hold position is significantly different for rising versus falling stimuli for the same absolute position for the neuron in Fig. 5A. In contrast, the response to hold positions for neuron in Fig. 5B consistently returned to the baseline (Fig. 5Biii). For both neurons, the preceding movement history influences response to hold positions. The neuron in Fig. 5A exhibits a strong asymmetry in responses to ventral and dorsal ramp movements.

History-dependence, which causes hysteresis, may be quantified as the area of the loop formed by the firing rate response to one cycle of positions starting from the most ventral position to the most dorsal position and back. Thus, the neuron in Fig. 5A exhibits a large hysteresis in the steady-state firing response with an area of 49.5 Hz-deg (Fig. 5Aiv). In contrast, the neuron in Fig. 5B has lesser hysteresis, with area of 15.4 Hz-deg (Fig. 5Biv).

The second source of hysteresis is the adaptation time constant. The first neuron (Fig. 5Ai) adapted more slowly to angular velocity (τ_v_=11.4 s) and to angular position (τ_p_=18.5 s) (Fig. 5Ai), whereas the second neuron exhibited a slightly faster adaptation to both velocity and position (τ_v_=9.6 s, τ_p_=12.4 s) (Fig. 5Bi). Across 59 neurons, we see a wide range of hysteresis values ranging from 4.67 to 64.11 Hz-deg (Fig. 5C).

### JO neurons show position-dependent response to angular velocity

**Fig. 6.**
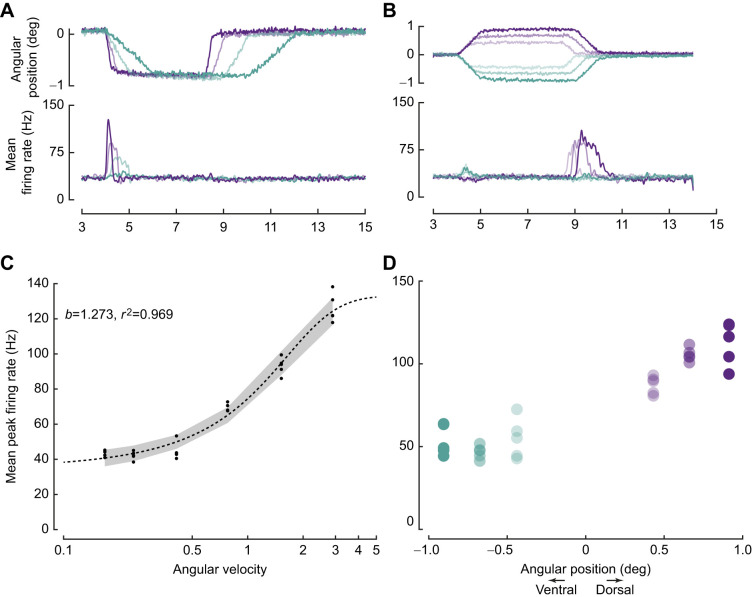
**Position-dependent response to velocity.** (A) Top: ramp-and-hold stimulus with variable ramp velocities (0.2 to 2.8 deg s^–1^). Bottom: corresponding responses in terms of mean firing rate (colour-coded). (B) Top: ramp-and-hold stimulus with variable hold positions (−0.9 to 0.9 deg s^–1^). Bottom: corresponding responses in terms of mean firing rate (colour-coded). (C) The logistic fit of peak firing rate versus angular velocity (deg s^−1^; *r*^2^=0.89), suggesting that the neuron encodes velocity. (D) Comparison of peak firing rate in response to ventral ramp movement from baseline positions (blue–green) and 0.9 deg (purple) at the same angular velocity of ca. 0.76 deg s^–1^.

In 20 neurons (*N*=18 moths), we provided ramp-and-hold protocols which recorded responses to both variable ramps and variable hold positions. Of these, one neuron responded strongly to ventral ramp movements, but remained largely inactive to dorsal ramp movements of the flagellum (Fig. 6A,B). Its peak firing rate in response to varying ramp velocity (but constant hold position) varied in a sigmoidal manner (Fig. 6C). In this neuron, the peak firing rates were greater when the flagellum moved from a more dorsal to zero position (Fig. 6B). The responses to ventral ramp movement from zero position was lower than the responses to ramp movement in the same direction but from more dorsal hold positions (Fig. 6D). (No statistical test was performed due to low power owing to small number of trials.) Thus, for this neuron, the peak firing rates in response to ramp movements depended strongly on the value of its hold position. In contrast to this neuron, in a majority of other 19 recordings, the peak firing rate for ventral ramp movement did not depend on their hold position.

## DISCUSSION

The experiments described in this study test the hypothesis that mechanosensory JOs encode angular position and angular velocity, which are the initial two components in the series decomposition of any time-varying mechanical stimulus. We performed intracellular recordings from axons of scolopidial units of the JO at the base of the antenna, while selectively varying the angular position and angular velocity of the flagellum using ramp-and-hold stimuli. Angular position was altered by moving the antennae to different hold positions, whereas angular velocity was varied by moving the antennae along ramp motions of different slopes. We recorded from 60 neurons from 32 moths. We also detected the actual movement of the antennae using a Hall-effect sensor placed very close to the location of the JO neurons.

Most neurons exhibited some baseline activity at the outset, likely owing to residual stimulus at resting position. In such cases, the response to a delivered stimulus was evident as a change relative to this baseline activity. In all neurons, the firing rate changed in response to antennal movement. These recordings show that JO neurons encode angular position and angular velocity. Most neurons exhibited a directional response to dorsal or ventral ramp movements, which in some neurons were different from their response to dorsal or ventral hold positions. In addition, the responses underwent rapid or slow adaptations to either angular velocity or position stimuli, or both. As a result of adaptation and directional sensitivity, JO neurons exhibited a range of hysteresis in response to hold positions.

### JOs respond to ventral ramp movement of the flagellum

A vast majority (56 out of 60) of the neurons responded to ventrad ramp movement of the antenna (Table 1), whereas their activity was inhibited by dorsal ramp movement. In the resting state and also when the antenna is positioned prior to onset of flight, the JO primarily experiences the weight of the flagellum in the direction of gravity, which suggests the intriguing possibility that the antenna may sense the direction of gravity. In many insects, including *Drosophila*, the antenna has previously been hypothesized as being a gravity sensor (Kamikouchi et al., 2009), but other data contradict this possibility (Kladt and Reiser, 2023 preprint).

The observation that the JO responds strongly to ventral ramp movement of the antenna is consistent with the hypothesis that the JO serves to detect gravity. Because the preparation described here was severely restricted to enable recordings from axons of single neurons, it was not possible within the scope of these experiments to test this function.

### JO neurons encode angular position and angular velocity

Our study showcases individual scolopidial units of the JO that encode both angular position and/or angular velocity. As the first two components in the series expansion of any arbitrary, time-varying stimulus, the encoding of position and velocity ensures that the JO can capture a major component of the antennal motion ranging from various low-frequency stimuli such as airflow or gravity (e.g. Gewecke, 1974; Kamikouchi et al., 2009; Yorozu et al., 2009; Roy Khurana and Sane, 2016; Suver et al., 2019; Natesan et al., 2019) to high-frequency stimuli such as antennal vibrations at wing beat frequency (Sane et al., 2007; Dieudonne et al., 2014). Although we have not explicitly tested for acceleration-sensitive units, we cannot exclude the possibility that some of the recorded units may exhibit sensitivity to acceleration. When insects including moths, honeybees and bumblebees are deprived of these stimuli through flagellar ablations, their flight control is compromised. The JO also plays a significant role in antennal positioning behaviour during flight in tethered hawkmoths (Natesan et al., 2019) and freely flying honeybees (Roy Khurana and Sane, 2016). Under constant airflow conditions, elimination or reduction of inputs to the JO by gluing the pedicel–flagellum joint eliminates the modulation of antennal positioning in both hawkmoths and bees. Angular position and velocity encoding in combination with the direction sensitivity of the JO neurons may be essential for antennal positioning. The inputs from the JO are shown to be critical for head stabilization, which in turn is essential for stable flight (Chatterjee et al., 2022). The ability of some neurons to encode both angular position and velocity (Figs 2 and 3) also points to their functional versatility.

### JO neurons are range-fractionated

Sensory neurons must contend with the trade-off between range and sensitivity. In many sensory organs including diverse eyes (Land and Nilsson, 2012), hearing systems (Göpfert and Hennig, 2016) and proprioceptive organs (Barth, 2019), this trade-off is resolved by distributing range over multiple units that comprise a sensory organ, while ensuring that each of those units is narrowly tuned to a specific frequency range. Previous research has shown range fractionation in frequency encoding by JO neurons in hawkmoths (Sane et al., 2007; Dieudonne et al., 2014) and, more recently, in flies (Patella and Wilson, 2018). Similarly, the mechanosensory neurons that constitute the femoral chordotonal organs in insect legs also exhibit range fractionation of leg angles over which individual neurons are sensitive to tibial movements (Matheson, 1992). The mechanosensory cercal hairs in crickets are range-fractionated by the length of the sensilla (Shimozawa and Kanou, 1984). Many studies in the field relating to mechanosensors leave open the possibility that the mechanical properties, geometry or location of the sensory units within an organ may play a key role in range fractionation, in addition to selective neuronal tuning properties.

In our experiments, the peak firing rate of JO neurons encoded angular velocity were modelled as a sigmoidal (logistic) function (Fig. 3C). Because the stimulus range in these experiments was only about 20-fold (∼0.2 to 4 deg s^−1^), we were unable to explore the possibility of power law relationships between the mechanical deflection of the antennae and the corresponding JO neuron responses, which typically require a stimulus range of at least two orders of magnitude. The practical difficulties in achieving such a range include the ability to deliver very fine and precise mechanical stimuli, as well as to deliver stimuli of a large magnitude. Such power law relationships have been previously suggested as being important in range compression by sensory neurons to enable single neurons to encode over a large range of stimuli (e.g. Thorson and Biederman-Thorson, 1974).

The various JO neurons characterized in this study vary in their sensitivity to delivered range of velocities, represented by varied range of slopes (Fig. 3C), which determines the range of velocities over which they encode. Consistent with previous data on *M. sexta* (Sane et al., 2007; Dieudonne et al., 2014), JO neurons in *D. nerii* also show range fractionation, which enables the antennae to serve a multimodal role ranging from sensing steady or low-frequency signals as required for airflow detection and gravity sensing (Kamikouchi et al., 2009) to sensing high-frequency signals as required for vestibular feedback (Sane et al., 2007).

### JO neurons display adaptation of response

Adaptation is a common feature across sensory neurons, interneurons and motor neurons. Here too, a power law (see Materials and Methods) with a fractional exponent has been proposed to capture adaptation, which may result from distributed relaxation processes (Thorson and Biederman-Thorson, 1974), distributed viscoelastic coupling (Chapman et al., 1979) and viscoelasticity of microtubules (Kuster et al., 1983). Power law adaptation has been shown at the stage of coupling between stimulus and the receptor (Loewenstein and Mendelson, 1965; Chapman et al., 1979), during transduction (Nakajima and Onodera, 1969a,b) and action potential generation (e.g. in femoral tactile spine in cockroaches, French, 1984a,b). In chordotonal organs, adaptation may occur at the transduction or spike generation stage (Field and Matheson, 1998).

The response of campaniform sensilla to similar ramp-and-hold protocols has been modelled as a nonlinear function of the instantaneous stimulus and an adapting variable (Szczecinski et al., 2021). This formulation simplifies the adaptation using first-order dynamics and predicts responses to various ramp-and-hold stimuli and response hysteresis in stair stimulus. However, that study assumed that the response to ramp-and-hold phases is monotonically decreasing.

Because we observed distinct adaptations to angular velocity and angular position stimuli, these two adaptations were separately characterized, using two exponents corresponding to the different time constants for velocity and position, respectively. The adaptation time constant was smaller for higher velocities of the stimuli and larger for lower velocities. As peak firing rate in response to angular velocity increases with angular velocity, the adaptation time constant may be related to the peak firing rate (Fig. S3). Firing rate may adapt faster following a higher firing rate owing to faster depletion of active channels involved in spike generation.

In sensory systems, adaptation performs a high-pass filtering of stimulus (Benda, 2021), thereby increasing the frequency threshold for activation. This process ensures sensitivity to small fluctuations riding over a sustained low-frequency stimulus. When combined with other non-linear phenomena, adaptation may help in ensuring stimulus selectivity and reduction of noise. For instance, adaptation to stimulus mean has been described in the fly auditory system, where the JO neurons correct for the background noise, thus remaining sensitive to courtship song (Clemens et al., 2018). In hawkmoths, we propose the hypothesis that adaptation helps keep the JO sensitive to minute perturbations when flying in a constant airflow condition (Natesan et al., 2019) or performing aerial turns (Sane et al., 2007; Dahake et al., 2018).

### Hysteresis in encoding position

Besides the encoding features introduced by adaptation, it is evident from the responses to stimuli in the stair protocol that adaptation – along with a directional response to stimuli – brings in a strong element of nonlinearity, including hysteresis, into sensory encoding. Hysteresis in a neural response is the dependence of neural activity on the history of stimulus, in addition to the current stimulus (Hatsopoulos et al., 1995). In the data presented here, the observed difference in steady-state firing rate in response to the same positions depended on the stimulus history, resulting from both adaptation and the directional response property of the JO neurons. Hysteresis behaviour has been reported in locust femoral chordotonal organs (Burns, 1974; Zill, 1985; Matheson, 1992), trochanteral campaniform sensilla in stick insects (Hofmann and Bässler, 1986) and cockroach tibial campaniform sensilla (Ridgel et al., 2000). Such hysteresis may occur because of asymmetries in the morphology of the sensor, biomechanical properties of the interstitial tissue, neural adaptation or a combination of these factors. Such history dependence is surprising because it means that the primary mechanosensory neurons provide feedback not only about the instantaneous stimulus state but also about how that state was attained. The implications of such history dependence for sensing of natural stimuli remain largely unexplored.

### Modelling the response of mechanosensory neurons

Various mathematical models have been used to describe the responses of mechanosensory neurons. Principal component analysis of descending interneurons that receive inputs from antennal hair sensilla at the scape–pedicel joint reveals movement sensitivity, posture sensitivity and direction sensitivity to be the first three principal components (Ache and Dürr, 2015). Fitting statistical models to measured response curves also reveals sensitivity to these components.

Control theoretic models that capture the response dynamics of antennal flagellar sensilla also reveal proportional and proportional derivative neurons (Mongeau et al., 2015) that correspond to encoding of wall position and velocity. A multiple linear regression model has been used to classify neurons of mechanosensory edge cells in lamprey that encode bending angle and bending rate as proportional and proportional derivative, respectively (Massarelli et al., 2017). Despite the differences in the sensory system and the modelling approaches, we see similarities in the components being encoded by the sensory neurons.

### The similarity of responses to femoral chordotonal organs and campaniform sensilla

The response properties of JO neurons are analogous to those observed in femoral chordotonal organs in insect legs. Similar combinations of position, velocity, acceleration and direction encoding have been demonstrated in femoral chordotonal organs of stick insects and locusts (Hofmann and Koch, 1985; Hofmann et al., 1985; Zill, 1985; Büschges, 1994). The stretch receptor organ in caterpillars of a hawkmoth (Simon and Trimmer, 2009) and flagellar sensilla on cockroaches (Mongeau et al., 2015) also encode position and velocity. Despite significant differences in structure and spatial distribution on the body, force encoding in campaniform sensilla in cockroaches (Ridgel et al., 2000; Zill et al., 2011) and encoding of leg bending in stick insects (Hofmann and Bässler, 1986) exhibit similar responses in terms of encoding angular velocity of movement or rate of change of force, encoding constant position or constant force, adaptation and hysteresis. These response characteristics are also comparable to responses in coxo-basal chordotonal organs in the legs of crabs (Bush, 1965) and mechanosensory edge cells in lamprey (Massarelli et al., 2017).

The similarities across various mechanosensory organs, despite their structural and functional diversity, suggest that the properties of the mechanosensory neurons are largely similar. However, specific encoding by these sensors may be determined by their location, biomechanics (Sane and McHenry, 2009; Barth, 2019) and associated mechanoreceptors.

The stimulus experienced by the sensory neuron is filtered by the physical structure of the mechanosensor (Sane and McHenry, 2009; Barth, 2019). A mechanical constraint on the movement causes a directional response in sensors, as seen in cercal filiform hairs in crickets (Gnatzy and Tautz, 1980). The orientation relative to the movement axis of tibial campaniform sensilla in cockroaches (Zill et al., 1981), wing campaniform sensilla in flies (Dickinson, 1992) and slit sensilla in spiders determines the strain patterns that are encoded (Barth, 2019). The mechanical properties determine the frequency that reaches the sensory organ, for example, cercal filiform hairs in crickets, the lateral line system in fish and tympanal organs in various insects (Sane and McHenry, 2009). A comparative study of the biomechanics of these mechanosensors, along with recordings of receptor potentials, will help us identify the key elements that sculpt the unique functionalities of these mechanosensors.

### Conclusions

This study provides evidence for encoding of angular position, angular velocity and direction of flagellar movement by the JO in hawkmoths. The JO neurons exhibit adaptation to either angular positions or velocities and a history-dependent response to angular positions. A vast majority of the neurons increase their activity in response to ventral movements, but their activity is suppressed for dorsal movements, which suggests direction sensitivity in the direction of gravity. The similarities in encoding properties to other mechanosensors across arthropods highlight the importance of biomechanics of these sensors that allow encoding of diverse mechanosensory stimuli.

## Supplementary Material

10.1242/jexbio.249342_sup1 Supplementary information
