# Supplementary material for: Encoding of antennal position and velocity by the Johnston's organ in hawkmoths
Source: J Exp Biol. 2025 May 2;228(9):jeb249342. doi: 10.1242/jeb.249342 (PMC12079665; doi:10.1242/jeb.249342)
Supplement: Supplementary information [file jexbio-228-249342-s1.pdf]

Supplementary Materials and Methods

Power law fit. A power law has been used to model the relation between flexion and extension velocity of the femoral chordotonal organ (Matheson, 1992) and the relation between the rate of change of forces on the tibial campaniform sensilla (Ridgel et al., 2000; Zill et al., 2011). Here we have used it to show the relation between peak firing rate and the angular velocity of movement. The power law is given by:

$$y(v) = a * v^k$$

which can be written as,

$$\log(y(v)) = k * \log(v) + \log(a)$$

where,

$y(v)$ = Peak firing rate, v = Angular velocity of flagellar movement,

$\log(a)$ = Intercept, and

k = Slope of the line on a log-log plot.

*Semilog fit.*The range of angular velocities and firing rates were speculated to be very narrow for using a power law. So, we attempted a semilog fit. The data was fit equally well by the following equation:

$$y(v) = k * \log(v) + c$$

where,

$y(v)$ = Peak firing rate, v = Angular velocity of flagellar movement,

c = Intercept, and

Journal of Experimental Biology • Supplementary information

k = Slope of the line on the semilog plot.

Multiple models explain the relationship between angular velocity and peak firing rate.

For a constant change in position, the peak firing rate increased with angular velocity of the flagellum. For instance, the neuron in **Fig 3A i** increased its peak firing rate by 1.5 folds for 16-fold change in its angular velocity (**Fig 3B i**). To capture the relation between peak firing rate and angular velocity, we fit the data to 4 different models (see methods).

Various studies have employed a power law to capture the relation between neural activity and the rate of change of a physical quantity (Matheson, 1992; Ridgel et al., 2000; Zill et al., 2011). Similar to these studies, in 19 out of 27 neurons (26 moths), peak firing rate and angular velocity followed a power law relationship (**Table S1, Fig. S1A**). Conventionally, at least 2 decades of data are employed to fit a power law. Since our recordings lacked a 2-decade change in angular velocity and firing rate, we also attempted to fit other models that capture the relation between the angular velocity and the peak firing rate response.

We find that the peak firing rate changes linearly with a decade change in angular velocity (**Fig. S1B**, semilog fit). Fits with r^2 values greater than 0.8 were considered to be good. In all, 19 out of 27 cells showed a velocity-sensitive response ($r^2 \geq 0.8$) (**Table S1, Fig. S1B**). We observed velocity encoding in most recorded neurons albeit with different slopes of the lines. The relation is captured the best in 19 out of 27 neurons by a logistic function, with more number of fits with $r^2 \geq 0.9$ (**Fig 3B, Table S1**). A linear fit underperforms with a good fit for only 14/26 neurons with $r^2 \geq 0.8$ (**Table S1,** and **Fig. S1C)**.

Journal of Experimental Biology • Supplementary information

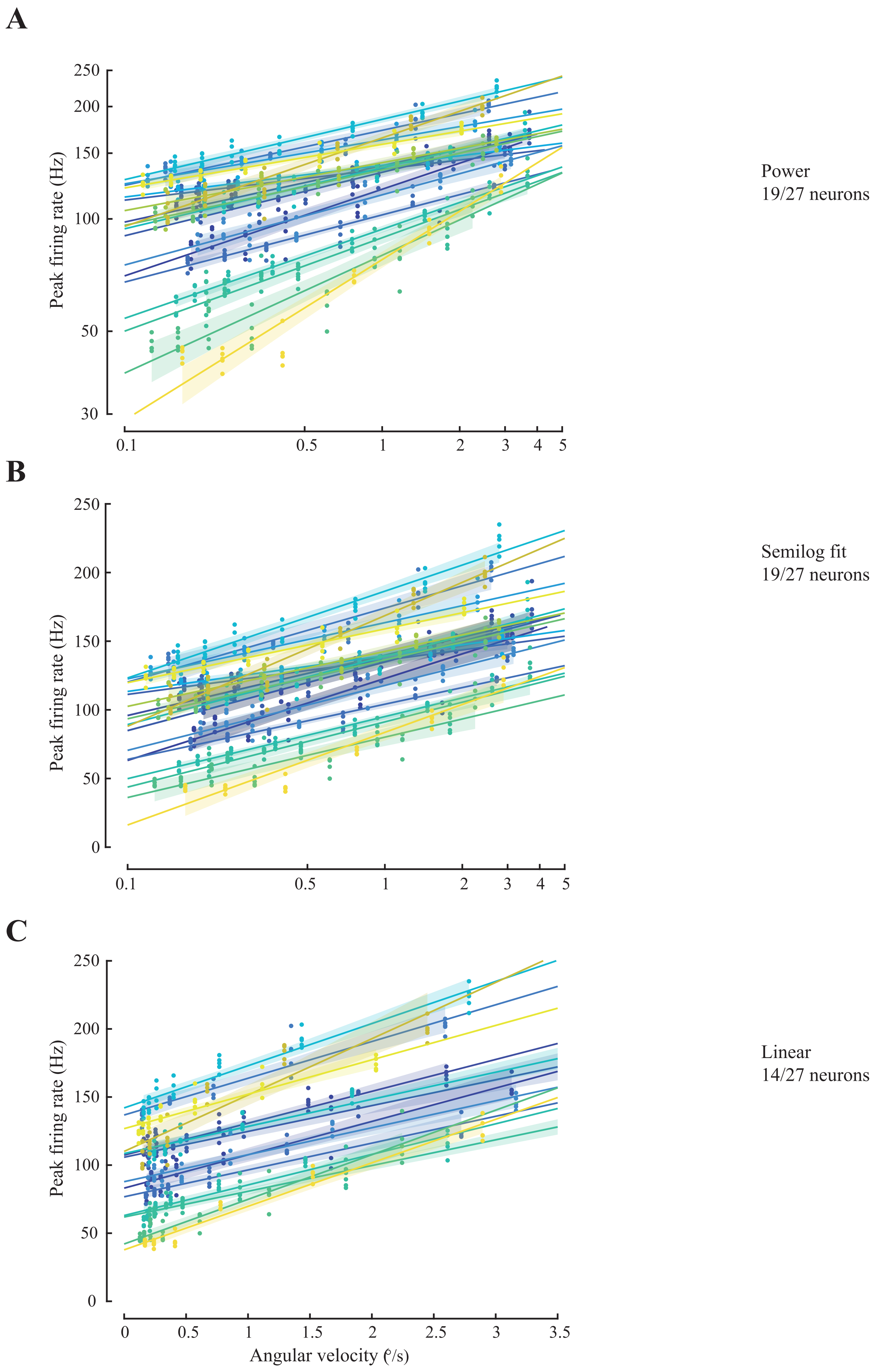

Fig. S1. Peak firing rate response versus angular velocity fit to other models. Response of 19 of 27 neurons explained (**A**) Power law; (**B**) Semi-log fit; (**C**) Linear fit. ($r^2 \geq 0.8$ for all). The shaded region around the fit is a 95% confidence interval around the fit.

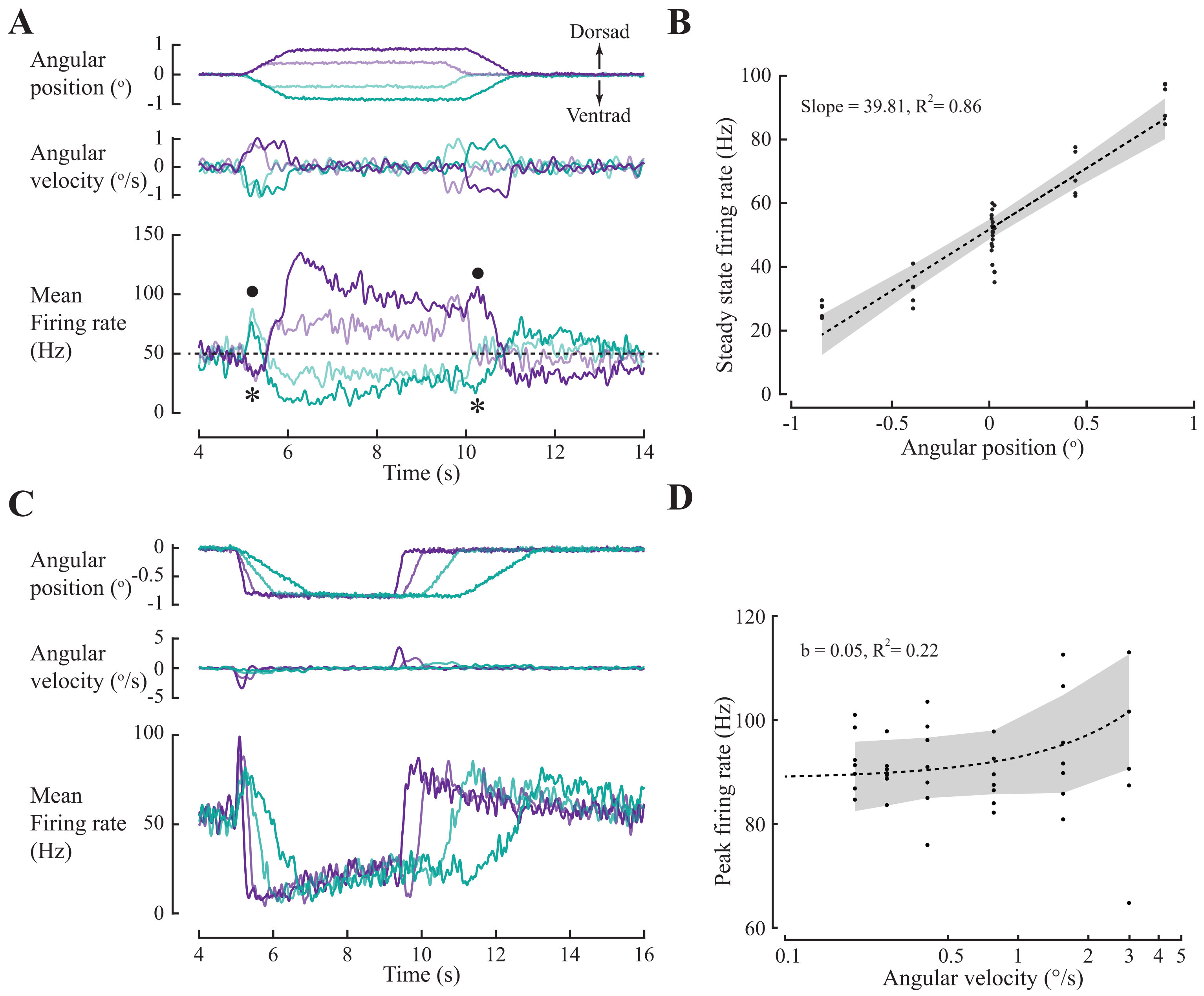

Fig. S2. Ventrad ramp-movement and position-sensitive neuron. (**A**) Same as Fig 2A. This neuron exhibits a brief increase in firing rate when a ventrad ramp movement is initiated (dot) and a brief dip in firing rate when movement is initiated in the dorsad direction (star). After this transient response, the response dynamics change within the ramp duration. (**B**) The steady-state firing rate linearly increases with the angular position of the flagellum ($r^2 > 0.8$). (**C**) Same as Fig 3A. (**D**) peak firing rate does not significantly vary at different velocities of movement. ($r^2 = 0.24$).

Journal of Experimental Biology • Supplementary information

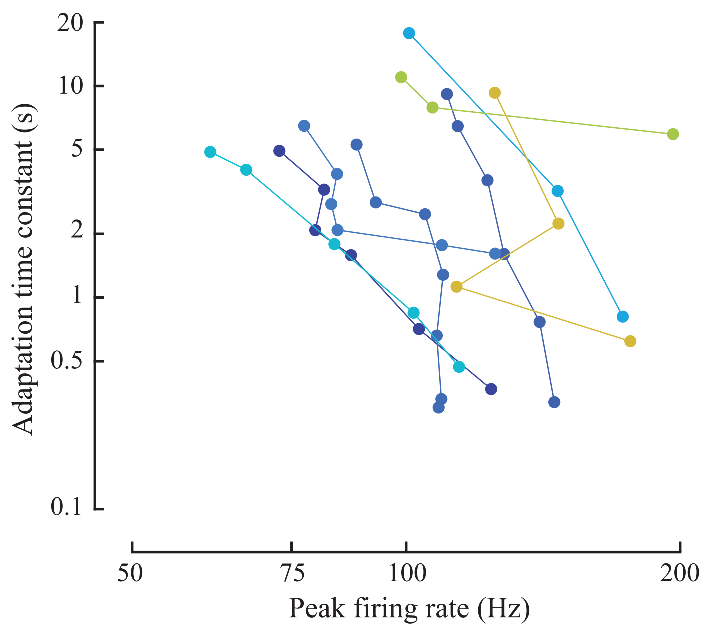

Fig. S3. Adaptation time constant versus peak firing rate elicited by different angular velocities. Same neurons as Fig 4E.

Journal of Experimental Biology • Supplementary information

Table S1. Comparison of goodness of fits for 4 models.

This table compares the rsquare values of the fits of 4 models that we used to find the relation between peak firing rate and velocity. The last 2 columns display the range of angular velocities tested and the corresponding range of mean peak firing rates. The rsquare values are color-coded as follows:

$r^2 \geq 0.9$
$r^2 \geq 0.8$
$r^2 \geq 0.75$

Neuron ID	Power law fit			Semilog fit			Logistic fit				Linear fit			Range	
	rsq	a	k	rsq	k	c	rsq	a	b	c	rsq	m	c	Velocity	FR (Hz)
2022-06-21_M2_N5	0.88	120.07	0.23	0.85	59.63	122.82	0.90	161.62	1.19	0.12	0.84	24.48	83.10	0.2 – 3.1	82 – 157
2022-06-22_M1_N1	0.83	138.92	0.15	0.80	43.91	139.79	0.89	195.26	0.61	-0.25	0.90	23.42	107.32	0.2 – 2.6	109 – 166
2022-07-06_M1_N1	0.33	95.18	0.07	0.34	14.31	95.33	0.41	99.22	2.71	-0.45	0.34	8.74	83.74	0.1 – 1.9	81 – 100
2022-07-07_M1_N1	0.87	133.30	0.17	0.86	49.98	134.87	0.87	173.63	0.84	-0.32	0.84	18.96	105.79	0.2 – 3.7	101 – 172
2022-07-07_M2_N3	0.81	135.80	0.08	0.80	24.93	136.17	0.82	148.19	1.31	-0.87	0.72	10.37	119.67	0.2 – 3	117 – 146
2022-07-07_M2_N5	0.02	113.99	0.02	0.02	4.19	114.04	0.13	115.00	13.34	-0.02	0.00	-0.06	111.56	0.2 – 3.1	104 – 119
2022-07-12_M1_N3	0.24	93.32	0.03	0.24	5.31	93.34	0.23	686.91	0.05	36.17	0.10	3.54	89.18	0.2 - 3	89 – 100
2022-07-13_M1_N1	0.96	102.50	0.18	0.94	40.19	104.10	0.97	124.95	1.34	-0.17	0.87	19.71	76.73	0.2 – 2.5	76 – 126
2022-07-14_M1_N1	0.89	172.73	0.15	0.85	53.86	174.10	0.94	218.22	0.83	-0.46	0.88	26.98	136.80	0.1 – 2.6	135 – 202
2022-07-18_M1_N4	0.84	116.02	0.19	0.82	47.28	117.79	0.88	154.98	0.85	-0.14	0.86	19.81	87.83	0.2 – 3.1	90 – 146
2022-07-20_M1_N3	0.84	162.84	0.12	0.84	40.97	163.50	0.84	179.55	1.75	-0.41	0.78	23.83	131.12	0.1 – 2.3	129 – 180
2022-07-21_M1_N1	0.84	139.17	0.08	0.83	26.13	139.52	0.85	155.09	1.04	-1.06	0.79	11.73	121.82	0.2 – 2.8	120 – 152
2022-07-22_M1_N1	0.93	184.84	0.16	0.92	63.19	186.42	0.94	236.15	0.90	-0.31	0.92	30.96	142.01	0.2 – 2.8	140 – 223
2022-07-23_M1_N3	0.92	137.16	0.16	0.91	49.62	138.88	0.93	181.16	0.77	-0.33	0.90	19.91	108.51	0.2 – 3.6	109 – 176
2022-07-25_M1_N1	0.97	93.81	0.24	0.96	45.22	95.10	0.95	119.98	1.39	0.09	0.89	22.44	62.99	0.2 – 2.6	59 – 116
2022-08-04_M1_N1	0.87	89.03	0.25	0.83	47.51	91.24	0.92	184.27	0.42	1.61	0.92	18.90	61.88	0.2 – 3.6	62 – 131
2022-08-09_M1_N2	0.87	79.89	0.32	0.81	43.96	80.12	0.93	157.59	0.82	1.18	0.91	32.76	42.08	0.1 – 2.2	46 – 117
2022-08-09_M1_N4	0.87	135.25	0.15	0.87	42.88	136.37	0.87	153.17	1.86	-0.23	0.77	21.70	105.31	0.1 – 2.5	100 – 154
2022-08-10_M2_N1	0.78	130.90	0.08	0.78	22.15	131.16	0.75	147.38	0.85	-1.35	0.73	11.70	115.02	0.1 – 2.6	113 – 145
2022-08-11_M1_N6	0.85	141.48	0.13	0.85	40.01	142.55	0.85	159.74	1.64	-0.36	0.73	17.92	115.39	0.1 – 2.7	108 – 159
2022-08-17_M2_N2	0.93	164.81	0.24	0.93	80.53	168.59	0.95	202.05	1.86	0.10	0.85	41.37	110.07	0.1 – 2.4	105 – 200
2022-08-22_M1_N1	0.01	149.90	0.07	0.01	21.94	150.10	0.15	2683.17	0.08	38.09	0.15	10.53	134.24	0.2 – 2.9	119 – 178
2022-08-23_M1_N3	0.42	101.17	0.04	0.42	8.30	101.21	0.56	100.33	13.15	-0.06	0.41	5.26	94.65	0.1 – 1.9	92 – 104
2022-08-23_M1_N8	0.22	65.23	0.04	0.22	5.22	65.26	0.19	67.39	1.38	-1.50	0.26	3.09	61.23	0.1 – 2.2	60 – 68
2022-08-24_M1_N4	0.95	77.80	0.43	0.89	67.48	83.61	0.97	133.00	1.27	0.81	0.96	31.95	37.78	0.2 – 2.9	42 – 126
2022-09-28_M1_N1	-0.01	109.49	0.02	-0.01	4.89	109.48	0.21	849.44	0.04	46.48	0.20	3.98	104.78	0.1 – 2.3	103 – 117
2022-09-29_M1_N1	0.90	158.43	0.12	0.89	39.13	158.93	0.90	181.12	1.21	-0.58	0.86	25.26	126.78	0.1 – 2	124 - 175

rsq ≥ 0.8	19			19			19				14				

Table S2. List of the neurons and their responses. Neurons used in figure are indicated in the last column of the table. In the cells, 0 : False, 1 : True, ? : indicates that the response was ambiguous.

Protocols tested	Cell ID	Position sensitive	Velocity sensitive	dorsad ramp movement	Ventrad ramp movement	Dorsad hold positions	Ventrad hold positions	Hysteresis (area of the loop)	Neuron used in Figure
Stair, Ramp and Step protocols	07-12_M1_N3	1	0		1	1		39.27	Figure 4C,D, Suppl. Figure 2A
	07-13_M1_N1	0	1		1			17.23	Figure 1C
	07-14_M1_N1	0	1		1			19.83	
	07-18_M1_N4	1	1		1	1		8.96	Figure 2 and 3
	07-20_M1_N3	1	1		1		1	44.99	
	07-21_M1_N1	0	1		1			26.07	
	07-22_M1_N1	0	1		1			12.31	Figure 5A
	07-25_M1_N1	0	1		1			13.15	
	08-04_M1_N1	1	1		1		1	7.55	
	08-09_M1_N2	0	1		1			4.67	
	08-09_M1_N4	0	1		1			15.55	
	08-10_M2_N1	0	0		1			64.11	
	08-11_M1_N6	0	1		1			24.58	
	08-17_M1_N1	0	0		?	1		24.91	
	08-17_M2_N3	0	0	?	?			18.53	Suppl figure 2B
	08-23_M1_N3	0	0		1			37.00	
	08-23_M1_N8	0	0		1			22.24	
	09-28_M1_N1	0	0		1			10.32	
	10-14_M1_N1	0	0		1			20.57	
Stair and Ramp	06-21_M2_N5		1		1			20.46	

Journal of Experimental Biology • Supplementary information

	06-22_M1_N1		1		1			22.84	
	07-06_M1_N1		0		1			11.67	
	07-07_M1_N1		1		1			20.43	Figure 5B
	07-07_M2_N3		1		1			7.24	
	07-07_M2_N5		0		1			27.07	
	07-23_M1_N3		1		1			14.81	
	08-17_M2_N2		1		1			49.69	
	08-22_M1_N1		0		1			7.32	
	09-29_M1_N1		1		1			33.45	
Ramp and Step	08-24_M1_N4	0	1		1				Figure 6
Only stair	06-21_M2_N1				1			8.62	
	06-21_M2_N4				1			17.39	
	07-01_M1_N1				1			25.78	
	07-01_M1_N2				1			35.67	
	07-05_M1_N1				1			20.23	
	07-05_M1_N4				1			16.45	
	07-06_M1_N2				?			9.44	
	07-07_M2_N2				1			8.01	
	07-12_M1_N2				1			16.12	
	07-12_M1_N4				1			8.57	
	07-18_M1_N1				1			35.33	

Journal of Experimental Biology • Supplementary information

	07-18_M1_N5				1			7.40	
	07-18_M1_N6				1			20.64	
	07-18_M1_N7				1			5.07	
	07-20_M1_N5				1			18.55	
	07-21_M1_N2				1			24.20	
	08-03_M1_N1				1			39.13	
	08-03_M1_N2				1			28.26	
	08-03_M1_N3				1			22.59	
	08-09_M1_N1				1			20.04	
	08-10_M2_N2				1			15.23	
	08-10_M2_N5				1			5.63	
	08-23_M1_N5				1			10.46	
	08-24_M1_N3				1			34.03	
	08-24_M1_N5				1			17.34	
	08-24_M1_N7				1			23.13	
	09-15_M1_N4				1			7.96	
	09-16_M1_N1				1			40.06	
	09-28_M1_N3				?			10.55	
	10-04_M1_N2				1			23.84	

References

Matheson, T. (1992). Range fractionation in the locust metathoracic femoral chordotonal organ. *J. Comp. Physiol. A* **170**, 509–520.

Ridgel, A. L., Frazier, S. F., DiCaprio, R. A. and Zill, S. N. (2000). Encoding of forces by cockroach tibial campaniform sensilla: implications in dynamic control of posture and locomotion. *J. Comp. Physiol. A.* **186**, 359–74.

Zill, S. N., Büschges, A. and Schmitz, J. (2011). Encoding of force increases and decreases by tibial campaniform sensilla in the stick insect, Carausius morosus. *J. Comp. Physiol. A* **197**, 851–867.

Journal of Experimental Biology • Supplementary information
